# Anti-angiogenic alternatives to VEGF blockade

**DOI:** 10.1007/s10585-015-9769-3

**Published:** 2015-11-30

**Authors:** Kabir A. Khan, Roy Bicknell

**Affiliations:** Angiogenesis Laboratory, Institute for Biomedical Research, School of Cardiovascular Research, College of Medical and Dental Sciences, University of Birmingham, Birmingham, UK

**Keywords:** Angiogenesis, Anti-angiogenesis, Cancer, Tumour, Therapy

## Abstract

Angiogenesis is a major requirement for tumour formation and development. Anti-angiogenic treatments aim to starve the tumour of nutrients and oxygen and also guard against metastasis. The main anti-angiogenic agents to date have focused on blocking the pro-angiogenic vascular endothelial growth factors (VEGFs). While this approach has seen some success and has provided a proof of principle that such anti-angiogenic agents can be used as treatment, the overall outcome of VEGF blockade has been somewhat disappointing. There is a current need for new strategies in inhibiting tumour angiogenesis; this article will review current and historical examples in blocking various membrane receptors and components of the extracellular matrix important in angiogenesis. Targeting these newly discovered pro-angiogenic proteins could provide novel strategies for cancer therapy.

## Introduction

Angiogenesis is the growth of new blood vessels from existing ones, it is an integral part of tumour progression and metastasis and is one of the original proposed hallmarks of cancer [1]. The main focus of anti-angiogenic strategies to date has been on the blockade of pro-angiogenic growth factors, the most important of which are the vascular endothelial growth factor (VEGF) proteins. The first blocking antibodies against VEGF were created by Genentech [2], and were later humanised as bevacizumab which became the first anti-angiogenic treatment gaining FDA approval. Anti-VEGF therapies have been reviewed extensively and will not be discussed here [3].

Many problems exist with VEGF inhibition therapy such as acquired resistance, due to the tumour microenvironment switching to utilise other pro-angiogenic growth factors such as fibroblast growth factor-2 (FGF2) [4]. Another caveat in anti-VEGF therapies is the promotion of metastatic and invasive cancer phenotypes seen in multiple tumour models [5, 6]. There is also emerging evidence that VEGF is not only a requirement for active angiogenesis but also normal vascular homeostasis through autocrine signalling and VEGF blockade can have negative effects [7]. The requirement of VEGF in non-angiogenic normal adult tissue function has also been reported, such as VEGF receptor activation leading to secretion of pro-inflammatory and pro-thrombogenic molecules from endothelial cells (ECs) in Wiebel-Palade bodies [8].

There is therefore a growing need for alternative strategies to halt the angiogenic process; one strategy is by inhibiting key protein–protein interactions other than VEGF that are important in angiogenesis. Anti-angiogenic agents that inhibit enzyme function such as receptor tyrosine kinase inhibitors will not be discussed here but have been extensively reviewed elsewhere [9]. This article will focus on current attempts and exciting new strategies in interfering with key extracellular protein–protein interactions as potential therapies against cancer.

## Fibroblast growth factors (FGFs)

FGFs are a family of growth factors that bind to membrane bound tyrosine kinase FGF receptors. FGF1, FGF2, and their receptors FGFR1 and FGFR2 are the main FGF molecules involved in angiogenesis, resulting in endothelial proliferation, migration and differentiation [10]. As mentioned previously FGF2 has been shown to be an important mediator of VEGF therapy resistance and this has been demonstrated clinically in patients with colorectal cancer treated with bevacizumab [11].

FGFs were first shown to be a targetable component of tumour angiogenesis in a study which utilised adenoviral mediated expression of a soluble form of the extracellular domain (ECD) of FGFR2 fused to an Fc tag. This acts as an FGF trap inhibiting the growth factor binding to cell bound FGF receptors, leading to reductions in pancreatic tumour formation in Rip1Tag2 mice [12]. A more recent version of this FGF trap was specifically engineered to have high binding affinity with FGF2 and was administered as a recombinant protein; this showed an anti-tumour effect in two different xenograft models [13]. A recent monoclonal antibody against FGF2 (GAL-F2) has shown promising anti-angiogenic and anti-tumour effects on a range of different hepatocellular carcinoma xenografts, and its effects could be increased by VEGF blockade [14]. These antibodies have been licensed to Roche for clinical development, highlighting their potential.

The discovery of a natural FGF2 antagonist named pentraxin-related protein 3 (PTX3) which inhibits FGF2-FGFR interactions has been utilised to create PTX3 derived peptides which can inhibit FGF2 dependent angiogenesis in vitro and in vivo [15]. Recombinant PTX3 or synthetic peptides could also inhibit tumour growth in prostate cancer models [16]. More recently PTX3 has been used to design a small molecule that can act as an extracellular inhibitor of FGF2 binding [17]. This inhibitor reduced tumour growth in syngeneic tumours and human xenografts when administered orally or by intraperitoneal injection.

FGF2 is normally present in high levels but is sequestered in the extracellular matrix (ECM) through binding to heparan sulphate containing proteins [18]. FGF binding protein (FGF-BP) is secreted by multiple tumours and can liberate FGF2 from the ECM [19]. The importance of this protein interaction is shown with siRNA knockdown of *FGF*-*BP* resulting in anti-tumour effects in colon carcinoma; this provides another possible target for anti-angiogenic therapy [20].

Dual blocking of VEGF and FGF2 has been achieved with the use of a fusion protein containing peptides of both VEGFA and FGF2, this fusion protein was used to vaccinate tumour bearing mice [21]. Tumour growth and tumour angiogenesis were both impaired, most likely due to the high titer of antibodies being raised against VEGF and FGF2 that could be detected in the blood.

## Platelet derived growth factors (PDGFs)

PDGFs are growth factors of which there are four members (PDGFA, B, C and D), these form homodimers or heterodimers which are essential for activation of the dimeric PDGF receptors of which there are two (PDGFRα and β) [22]. The main pro-angiogenic components are PDGFB and the pericyte expressed receptor PDGFRβ which are important for pericyte-endothelial interactions [23]. A PDGFB binding DNA aptamer (AX102) has been generated which inhibits PDGFB-PDGFRβ interactions; this aptamer could cause pericyte loss and vascular regression in syngeneic mouse tumour models, although this did not affect overall tumour growth it provided a strategy that could be used in combination with other anti-angiogenics [24]. Indeed, a later study used ovarian cancer xenografts to show that AX102 in combination with bevacizumab could enhance the anti-tumour effect of bevacizumab alone [25].

## Placental growth factor (PlGF)

PlGF is part of the VEGF family, operates through VEGFR1 homodimers and is not essential for normal angiogenesis (PlGF deficient mice are viable and healthy) but is important in pathological angiogenesis [26]. There have been conflicting results involving PlGF blockade, some studies have shown anti-tumour activity against VEGFR inhibited tumours in mice [27]. Whereas others have shown PlGF blockade has limited anti-angiogenesis action in vitro [28] and recent in vivo studies have argued against its ability to reduce tumour angiogenesis [29]. These effects are likely to be context dependent and PlGF blocking antibodies are currently undergoing clinical trials.

## Angiopoietins

The angiopoietins, of which there are four members (Ang1-4), are growth factors which bind to the tyrosine kinase receptors Tie1 and Tie2 [30]. The role of angiopoietins in angiogenesis is somewhat complex, Ang1 is a strong agonist and Ang2 a partial agonist of Tie2 [31], in the presence of high levels of Ang1, Ang2 can act as an antagonist to Ang1-Tie2 interactions [32]. Ang1 is thought to mainly stabilise and protect the existing vasculature [33] whereas Ang2 prepares endothelium for active angiogenesis maintaining a “plastic” state [34]. Ang2 can also increase endothelial cell (EC) migration and sprouting in a Tie2 independent manner through integrin signalling [35].

Ang2 is mainly expressed during development and in areas of the adult that undergo vascular remodelling [36]; it is also highly expressed in cancer. In the tumour setting a pattern emerges where the ratio of Ang1 to Ang2 is increased in favour of Ang2, supporting active angiogenesis [37]. These are the main reasons for the drive to develop anti-angiogenic agents targeting the Ang2-Tie2 interaction. There have been two main methods in blocking this interaction, namely peptide or antibody based approaches. The drug trebananib is a peptibody (peptide-Fc fusion) that contains two peptides per molecule which can block Ang2 and Ang1 from interacting with Tie2 receptor. Trebananib inhibits colorectal xenograft tumour growth and rat corneal vascularisation [38]. Unfortunately trebananib has yielded disappointing results in a phase III clinical trial for ovarian cancer [39]. More specific inhibitors of Ang2 have been developed including a Tie2-ECD-Fc ligand trap [40]. In this study directed evolution using B cell somatic hypermutation was applied to create a ligand trap that preferentially bound to Ang2 and not Ang1. This application resulted in a great advance in selective Ang2 inhibitors, but more importantly the method developed here could be used in a whole host of different settings to create higher affinity and specificity antibodies or ligand traps.

Blocking antibodies against Ang2 have been developed separately by Medimmune (MEDI3617) and Regeneron (REGN910) [41, 42]. The use of these antibodies inhibited xenograft tumour growth in both cases and each effect was enhanced with VEGF blockade, these agents are both undergoing phase I clinical trials. The success seen with inhibition of both Ang2 and VEGF has led to the development of a bispecific antibody by Roche which can block both of these growth factors [43]. In a wide range of different tumour xenograft models, this bispecific antibody showed anti-angiogenic and anti-metastatic properties and could even cause tumour regression when used in combination with chemotherapy. There is emerging evidence that suggests upregulation of Ang2 in some cancers is involved in tumour resistance to anti-VEGF therapies [44] therefore combating both of these protein interactions seem to be a reasonable approach.

## Notch receptors and ligands

The evolutionarily conserved Notch signalling pathway in mammals involves four Notch receptors (Notch1-4) and five Notch ligands (Jagged 1 and 2 and Delta-like ligands Dll1, Dll3 and Dll4) [45], Notch 1, 2 and 4 and all ligands except Dll3 are expressed in ECs [46]. Notch signalling is vital for angiogenesis; this can be demonstrated in mice with endothelial specific *Notch1* deletion leading to embryonic lethality, due to defects in vessel maturation and angiogenesis whereas vasculogenesis is unaffected [47]. Notch signalling is vital for sprouting angiogenesis and the formation of endothelial tip and stalk cells. Upon VEGF stimulation tip cells begin to upregulate notch ligands such as Dll4, which then bind to notch receptors on adjacent ECs. The activation of notch signalling leads to downregulation of VEGF receptor 1 and 2 (VEGFR1 and 2) and formation of a stalk cell phenotype [48, 49]. Dll4 has been found to be upregulated in the vessels of tumour xenografts and also in the vessels of human tumours suggesting a good target for anti-angiogenic agents [50, 51].

Notch protein interactions have been successfully targeted numerous times by different methods. Dll4 blockade using monoclonal antibodies caused ECs in vitro and in vivo to display increased sprouting and increased proliferation, most likely due to the lack of inhibitory cues from a tip cell, therefore all ECs under VEGF stimulation become of the tip cell phenotype. This Dll4 inhibition was anti-angiogenic and showed anti-tumour effects in six different tumour models [52]. Notch-Dll4 protein interaction inhibition was also achieved by use of a soluble Dll4 ECD fused to an Fc tag (Dll4-ECD-Fc) in two separate studies, this approach phenocopied effects on tumour angiogenesis seen with the antibody blocking strategy [53, 54]. Despite increasing vessel branching and sprouting, the anti-angiogenic effects seen with inhibition of the Notch pathway in the above examples, are likely due to formation of non-functioning vasculature which leads to poor perfusion and hypoxia in tumour tissue [55]. Targeting the notch pathway using Notch1 specific antibodies has also been shown to have similar anti-angiogenic and anti-tumour effects in two different xenograft models [56]. Soluble versions of the Notch1 receptor have also been developed, utilising the whole of the Notch1 ECD fused to an Fc tag (Notch1 decoy) this had anti-angiogenic effects in mouse tumour xenografts [57]. More recently Notch decoys containing domains that bind to Jagged, Dll1/Dll4 or both have been created [58]. The Dll1/Dll4 binding decoy causes vessel hypersprouting in vitro, this fits with the already proposed model of Dll1 and Dll4 Notch signalling resulting in inhibitory signals inducing cells into a stalk cell phenotype. This decoy also has anti-tumour effects most likely due to mechanisms already discussed with Dll4 blockade. The notch decoy which blocks Jagged1 and Jagged2 reduced EC sprouting in vitro and retinal angiogenesis in vivo. This decoy also reduced tumour growth due to decreased tissue perfusion, reduced coverage of pericytes and reduced sprouting. The authors propose a mechanism where Notch1-Jagged signalling is pro-angiogenic by downregulating expression of the decoy soluble VEGFR1 receptor and is important for endothelium to associate with pericytes aiding in vessel maturation.

Although blocking Dll4 has shown anti-tumour responses in these pre-clinical models, the aberrant effects of chronic Dll4 inhibition have also been investigated. Sustained treatment with anti-Dll4 antibodies result in abnormal liver pathology and can give rise to vascular neoplasms in various species including monkeys, rats and mice [59]. Similarly low frequency genetic loss of *Notch1* in adult mice leads to increased endothelial proliferation and the formation of vascular tumours [60]. Nevertheless humanised anti-Dll4 antibodies (demcizumab) are currently undergoing clinical trial evaluation in various tumour types. Alternative approaches could include specifically inhibiting Notch-Jagged protein interactions without inhibiting Notch Dll1/Dll4, as inhibiting in this way does not cause hypersprouting and hyperproliferation which is the likely mechanism leading to vascular neoplasms.

## Integrins

Integrins consist of α and β subunits which dimerise to mainly bind components of the ECM and elicit signal transduction events. In endothelium the major integrins and the most targeted are α5β1, αvβ3 and αvβ5 which are upregulated during active angiogenesis [61, 62]. α5β1 and αvβ5 bind to fibronectin and vitronectin respectively, whereas αvβ3 has a larger range of interacting proteins, including fibronectin, vitronectin, and fibrinogen among others [63]. These integrins bind via the Arginine-Glycine-Aspartic acid (RGD) motif which was first discovered to be important in fibronectin [64]. αvβ3 is required for angiogenesis induced by FGF2 or TNFα and αvβ5 is required for VEGF and TGFα activation, cyclic RGD peptides or antibodies against either integrin could block growth factor induced angiogenesis [65].

The findings that αvβ3 is highly expressed on activated endothelium during angiogenesis and has high expression on tumour vasculature gives it targeting potential [66, 67]. Humanised anti-αvβ3 antibodies (Vitaxin or etaricizumab) have yielded promising preclinical and phase I results [68, 69] but unfortunately have had little effect on disease progression in phase II trials in melanoma [70]. A cyclic RGD peptide (cilengitide) that blocks both αvβ3 and αvβ5 protein interactions has shown preclinical success in mouse models of breast cancer [71, 72]. In a recent phase III trial of newly diagnosed glioblastoma, cilengitide was combined with chemoradiotherapy which resulted in no significant benefit and the subsequent suggestion by the authors to halt further cilengitide development in its current form for cancer therapy [73]. The inhibition of αvβ3 and αvβ5 integrins has so far been disappointing clinically and it is unclear whether inhibiting these integrins will yield significant clinical benefit. It is interesting to note that pro-tumour and pro-angiogenic effects are seen with RGD peptide inhibitors of αvβ3 and αvβ5 at low concentrations in mouse models [74]. More recently cilengitide has been used in combination with the calcium channel blocker verapamil to promote tumour vascularisation in lung and pancreatic mouse tumours [75]. This allowed better perfusion and delivery of chemotherapeutic agents resulting in reductions in tumour growth and metastasis.

Antibodies blocking α5β1 (volociximab) can induce apoptosis of proliferating ECs in vitro and could inhibit choroid vascularisation in cynomolgus monkeys [76]. As volociximab does not recognise murine α5β1, rat anti-mouse monoclonal antibodies have been generated which have anti-tumour effects in mouse tumour models [77]. Volociximab is currently undergoing further trials, but Phase I trials in non-small cell lung cancer have shown partial response in some patients [78].

## VE-cadherin

VE-cadherin is an endothelial specific adhesion molecule found at cell–cell contacts where it can bind to other VE-cadherins on neighbouring cells forming adherens junctions (AJs) [79]. VE-cadherin gene expression has been shown to be upregulated in tumour angiogenesis and is upregulated in response to FGF2 [80]. Monoclonal antibodies against VE-cadherin have shown reductions in tumour growth without causing vascular permeability [81]. Interestingly an antibody that specifically binds to a region of VE-cadherin that is only exposed when ECs are undergoing neoangiogenesis has been developed, this antibody could still disrupt AJs and offers a way of inhibiting VE-cadherin function in active angiogenesis [82]. The first three cadherin domains of VE-cadherin have displayed anti-angiogenic properties in a HUVEC tube formation assay and a colon carcinoma xenograft model [83]. This soluble VE-cadherin ECD most likely disrupts VE-cadherin homotypic binding and endothelial cell–cell contacts.

## Ephrins and Eph receptors

The tyrosine kinase Eph receptors consist of 15 different members which bind differentially and promiscuously to 9 membrane bound ligands to elicit a range of effects such as migration, proliferation, survival and tissue patterning [84]. Signalling events can occur through the Eph receptor (forwards) or through the ephrin ligand (reverse) [85]. In angiogenesis two main protein interactions take place, EphA2-ephrinA1 and EphB4-ephrinB2, these are the most studied and most targeted [86].

EphrinA1 is expressed at sites of vasculogenesis in the developing embryo [87] and is also expressed on the vasculature and on tumour cells of mouse xenografts and various human tumours, including those of breast cancer patients [88]. EphrinA1 expression and subsequent EphA2 activation has been shown to be upregulated by VEGF. Blocking this protein interaction using an EphA2-ECD-Fc decoy reduced VEGF induced but not FGF2 induced EC function [89]. The use of EphA2-ECD-Fc has been shown to reduce tumour angiogenesis in vivo in Rip1Tag2 pancreatic and 4T1 breast tumour models; it also had inhibitory effects on bovine microvascular cells in vitro but not on the 4T1 tumour cells in culture demonstrating vasculature specific effects [90]. The EphA2-ECD-Fc also had anti-tumour and anti-angiogenic effects on human xenografts, and in orthotopic models of pancreatic cancer [91].

EphB4-ephrinB2 interactions have been implicated in angiogenesis and vasculogenesis, ephrinB2 is essential for correct artery formation and its receptor EphB4 correct vein formation. This process is dependent upon forward and reverse signalling of both proteins [92]. In tumour angiogenesis the expression of EphB4 on tumour cells has been shown to be important in interacting with ephrinB2 on ECs and promoting tumour angiogenesis [93]. Furthermore ephrinB2 reverse signalling is required for EC tip guidance by internalisation and subsequent activation of VEGFR2; ephrinB2 signalling deficiency results in decreased tip cell formation and is therefore an attractive target [94]. The most promising approach inhibiting this interaction so far involves a soluble EphB4 ECD conjugated to human serum albumin (EphB4-ECD-HSA), this has shown anti-angiogenic effects on pancreatic tumours in Rip1-Tag2 mice, which could be improved with Dll4-Notch blockade using Dll4-ECD-Fc [95]. EphB4-ECD-HSA can also have inhibitory effects on some tumour cells and has led to complete remission in bladder cancer xenografts with bevacizumab treatment [96]. EphB4-ECD-HSA is currently undergoing phase I clinical evaluation.

## CLEC14A

CLEC14A is a tumour endothelial marker upregulated in the vasculature of a range of different tumour types compared to healthy tissue [97]. Our group and Zanivan et al. have independently shown that CLEC14A binds to an endothelial specific ECM protein multimerin-2 (MMRN2) [98, 99]. siRNA knockdown of *CLEC14A* or *MMRN2* results in impaired angiogenesis in vitro [100, 97], furthermore both of these proteins have been shown to be upregulated with tumour progression in spontaneous mouse models [99]. These reasons make the CLEC14A-MMRN2 interaction an attractive one for anti-angiogenic targeting. We have recently identified a monoclonal antibody against CLEC14A that can inhibit it from binding to MMRN2. This blocking antibody has detrimental effects on angiogenesis in vitro in tube formation and spheroid sprouting assays, but more importantly this antibody can also disrupt tumour angiogenesis in a Lewis lung carcinoma (LLC) model leading to reductions in tumour growth [98]. Antibodies raised specifically against the C-type lectin domain of CLEC14A have also been shown to have anti-angiogenic effects; we hypothesise that these may also interrupt the CLEC14A-MMRN2 interaction [101].

## TEM8

TEM8 or ANTXR1 is an anthrax toxin receptor which has been identified as a tumour endothelial marker [102]. TEM8 has been shown to interact with the α3 subunit of collagen VI; this interaction partner was also found to be upregulated in tumour endothelium, suggesting that the interaction may be a target for anti-angiogenics [103]. TEM8 knockout mice develop relatively normally but display impaired angiogenesis in tumour xenografts leading to reduced tumour growth [104]. When TEM8 is blocked with monoclonal antibodies, this too results in reductions in tumour xenograft growth with melanoma showing the highest efficacies [105]. TEM8 blockade was most effective when combined with VEGF blockade and chemotherapy. The extracellular domain of TEM8 fused to an Fc tag (TEM8-ECD-Fc) also has anti-angiogenic effects and inhibits growth in tumour xenografts [106]. This is likely due to the TEM8-ECD-Fc binding to TEM8 ligands and inhibiting membrane bound TEM8 interactions.

## MCAM (CD146)

MCAM or melanoma cell adhesion molecule (CD146) is a VEGFR2 co-receptor, has implications in tumour angiogenesis and is found to be upregulated in a wide range of different cancers [107]. Mice deficient in endothelial MCAM develop normal vasculature but display defects in tumour growth [108]. A number of protein interactors have been identified for MCAM including the ECM protein laminin-411 [109]. More recently MCAM has been shown to interact with the neuronal guidance protein netrin-1, this interaction was shown as pro-angiogenic, enhancing EC proliferation, migration and tube formation [110]. A monoclonal antibody against MCAM could block this interaction and the interaction with VEGFR2. The same monoclonal antibody has previously been demonstrated to have anti-angiogenic and anti-tumour effects in xenograft models which could be enhanced with addition of bevacizumab [111]. Disrupting the MCAM-netrin-1 and MCAM-VEGFR2 interactions are the likely mechanisms of this effect, although the authors did not test whether the antibody disrupts MCAM binding to other known ligands.

## Endoglin

Endoglin or CD105 is a dimeric co-receptor for transforming growth factor-β (TGF-β) and is expressed on adult endothelium and some haematopoietic cells including proerythroblasts [112, 113]. Endoglin deficient mice die at embryonic day 11.5 due to defects in angiogenesis and vessel remodelling but display no defects in vasculogenesis [114]. Endoglin is highly expressed on proliferating endothelium including that of a range of human tumours [115]. It is upregulated in response to hypoxia and VEGF blockade, for these reasons endoglin poses another attractive target for therapies [116, 117]. Tumour xenografts treated with anti-endoglin monoclonal antibodies showed anti-angiogenic effects which could be enhanced with chemotherapy, this antibody was used to create a human chimeric antibody named TRC105 [118]. While TRC105 has been linked with antibody directed cell cytotoxicity (ADCC) [119], a recent study has shown TRC105 to inhibit BMP-9 binding to endoglin and the BMP receptor complex, resulting in inhibition of SMAD1 signalling leading to arrest in vessel formation, suggesting a possible mechanism of action [120]. TRC105 shows anti-angiogenic properties in vitro which is enhanced when combined with bevacizumab [121]. Phase I trials of TRC105 in combination with bevacizumab in various advanced solid tumours look to be promising, with some patients displaying reductions in tumour volume, further trials are underway [122].

## Advantages in protein–protein inhibition

### Higher specificity

If a protein interaction is targeted then both components will need to be expressed and important for pathological angiogenesis. A good example is the co-expression of CLEC14A and MMRN2. If MMRN2 is important for other vascular functions, which is highly likely, then by specifically inhibiting its interaction with CLEC14A that only appears to be important in neoangiogenesis, aberrant effects elsewhere are likely to be minimalized.

### Expression in tumour cells

While high specificity in tumour angiogenesis is a desired characteristic, this is not the case if the interaction is also important in tumour cells as seen with some Eph-ephrin and integrin interactions.

### Better tolerance

Many of the strategies in inhibiting protein interactions discussed in this review involve using human antibodies or decoys derived from human sequences, as these are biological agents they are likely to be better tolerated in patients and less likely to elicit an immune response.

### Ease of design

**Fig. 1:**
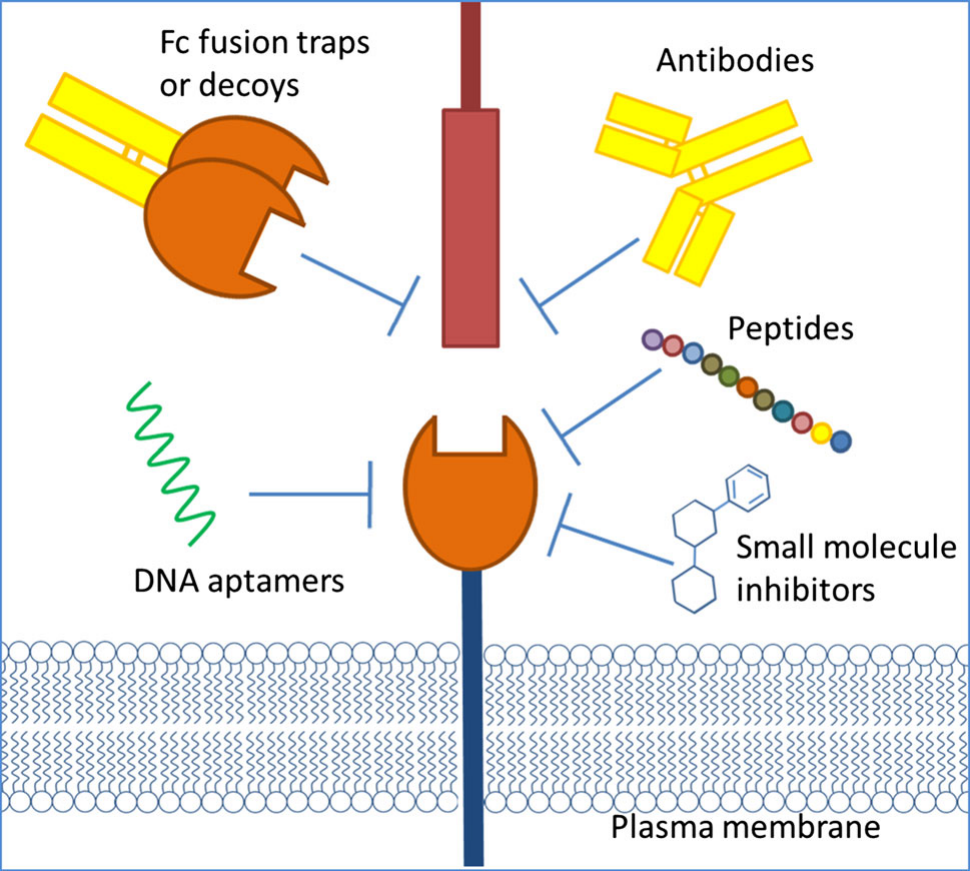
Strategies in disrupting protein–protein interactions. *Antibodies* against either protein (if possible raised against domains known to be involved in interaction). If the target protein is ubiquitously expressed then the Fc region of the monoclonal antibody can be mutated to block immune cell recognition, therefore only the blocking function of the antibody will likely remain. *Fc fusion traps or decoys,* by fusing the ECD of either the ligand or receptor to an Fc tag and producing a soluble version which can bind but elicit no signalling response. *Peptides or peptibodies* these would require a linear binding site to be determined which can then be synthesised as a peptide, alternatively high throughput screening of peptide libraries could be used. *Small molecule inhibitors*, this approach is more difficult and would most likely rely on a structure being solved of the protein interaction complex so molecules can be designed. Alternatively, high throughput screening could be used on libraries of drug compounds. *DNA or RNA Aptamers* that can inhibit protein interactions such as pegaptanib which binds to VEGFA 165 isoform [123]. Advantages include the ease and low cost of synthesis of such agents

When a potential pro-angiogenic protein interaction is revealed, the use of decoys or antibodies directed against either protein can be easier than screening small molecules that may inhibit a receptor or ligand function (discussed in Fig. 1). The fact that there are currently no specific TIE2 small molecule kinase inhibitors, but there are many protein based approaches in disrupting its interactions emphasises this point.

## Disadvantages in protein–protein inhibition

### Possible off target effects

In theory specificities may be increased using protein–protein interaction strategies as described above, in reality this is limited by the knowledge of a certain interaction pathway. It is important to note that some patients in the TRC105 trial displayed hypoproliferative anemia due to endoglin expression on proerythroblasts [122]. Some relatively new interactions with little known about them could be important for other functions within an adult resulting in off target effects. Furthermore inhibiting a certain receptor or ligand has the potential to disrupt interactions with other partner proteins that may be currently unknown. Therefore with more basic research into newly discovered angiogenic pathways, these important functions can be dissected. The need for good preclinical models is also key here, with careful attention being given to possible side effects or abnormalities occurring in other tissues other than tumours.

### Potential for resistance

As is seen with VEGF blockade, the potential for the tumour microenvironment to become resistant to certain therapies is high. As there are a large range of pro-angiogenic interactions and pathways it would be difficult and unfeasible to target them all. Hence the use of easily detectable biomarkers in cancer patients undergoing treatment will be of great benefit, to determine the best strategies for alternative therapies when and if resistance occurs.

## Emerging approaches

The majority of work in inhibiting protein interactions has been through the use of antibodies binding and blocking normal protein function. Indeed this strategy has had the most success clinically, with a large number of antibodies targeting various angiogenic factors entering clinical trials (Table 1). The future of antibody therapy will most likely involve the ability to bind more than one antigen. This has been seen with bispecific antibodies and more recently the creation of zybodies, that can bind up to five different targets by the use of peptides added to traditional antibody scaffolds [124]. This adds the capability to target multiple pro-angiogenic molecules using only one therapy, which would be advantageous as many of the discussed examples display increased efficacy when combined with VEGF blockade.

**Table 1:** Protein–protein disrupting agents and their preclinical and clinical progress

Anti-angiogenic agent	Interaction inhibited	Pre-clinical tumour models used	Anti-tumour effect	Clinical trials	References
FGF-trap FGFR2-ECD-Fc	FGF2-FGFR2	**G **Rip1Tag2, **AG **βHC13T pancreatic	An average of ~80 % reduction in tumour growth	None	[12]
GAL-F2 FGF2 antibody	FGF2-FGFR2	**XG **SMMC-7721, HEP-G2 and SK-HEP1 hepatocellular carcinomas	Tumour regression when combined with anti-VEGF	None	[14]
AX102 (Fovista) Anti-PDGFB aptamer	PDGFB-PDGFRβ	**XG OT** Skov3ip1 and HeyA8 ovarian cancer	65–88 % reductions in tumour growth in combination with bevacizumab	Phase III for age related macular degeneration	[25]
TB-403 Humanised PlGF antibody	PlGF-VEGFR1	**SG **B16 melanoma, **SG OT** Panc02 pancreatic and **SG **CT26 colon cancer	55–66 % reductions in tumour growth	Phase I solid tumours	[27]
Trebananib Ang1 and Ang2 binding peptide	Ang1/2-Tie1/2	**XG **A431 epidermoid, **XG **Colo205 colon carcinoma	Average of ~50 % reductions	Phase III ovarian cancer and various others	[38]
MEDI-3617 (Tremelimumab) Fully human Ang2 antibody	Ang2-Tie1/2	**XG **LoVo and Colo205 colon carcinoma, 786-0 renal carcinoma, HeyA8 ovarian cancer and PLCPRF/5 hepatocellular carcinoma	45–85 % reductions in tumour growth	Phase I metastatic melanoma	[41]
REGN910 (Nesvacumab) Fully human Ang2 antibody	Ang2-Tie1/2	**XG **A431 epidermoid, **XG ** PC3 prostate, **XG **Colo205 colon carcinoma	50–70 % reductions in tumour growth	Phase I solid tumours	[42]
Ang-2 VEGF CrossMab Human Ang2 and VEGF antibody	Ang2-Tie1/2 VEGF-VEGFR	**XG OT** KPL-4 breast cancer, **XG** Colo205 colorectal and a large range of patient derived cell lines	Complete tumour regression in combination with docetaxel	None	[43]
OMP21M18 (Demcizumab) Humanised Dll4 antibody	Dll4-Notch	**XG** HM7 colon, **XG** Colo205 colon, **XG** Calu6 lung, **XG** MDA-MB-435 breast, **XG** MV-522 lung	50–70 % reductions in tumour growth enhancement seen in combination with anti-VEGF	Phase II ovarian, non-small cell lung and pancreatic	[52]
Dll4-ECD-Fc	Notch–Notch ligands	**XG** HT29 colon adenocarcinoma, **XG** KS-SLK Kaposi sarcoma	Average ~70 % reductions in tumour growth	None	[54]
Notch1-ECD-Fc	Notch–Notch ligands	**SG** Mm5MT breast cancer, **XG** NGP neuroblastoma	Average ~75 % reductions in tumour growth	None	[57]
MEDI-522 (Etaricizumab) Vitaxin Humanised anti αvβ3 monoclonal antibody	αvβ3-ligands	**XG** MCF7 breast cancer	80–90 % reductions in tumour growth some complete regressions	Phase II metastatic prostate and melanoma	[68]
Cilengitide RGD peptide	αvβ3-ligands	**XG** HBT 3477 breast adenocarcinoma	Complete tumour regression in 53 % of mice when combined with radioimmunotherapy	Phase III glioblastoma	[72]
Volociximab Chimeric anti α5β1 antibody	α5β1-ligands	**XG** A673 rhabdomyosarcoma, SVR angiosarcoma	40–60 % reductions in tumour growth	Phase I Non-small cell lung cancer	[77]
EphA2-ECD-Fc	EphaA2- EphA2 ligands	**XG OT** Colo357 pancreatic cancer	~50 % reductions in tumour growth	None	[91]
EphB4-ECD-HSA	EphB4-ephrinB2	**XG** 5637 bladder cancer	62 % reductions and complete regression when combined with bevacizumab	Phase I solid tumours	[96]
CLEC14A antibody Mouse antibody	CLEC14A-MMRN2	**SG** Lewis lung carcinoma	Average reductions of ~60 %	None	[98]
TEM8 antibody Chimeric antibody	TEM8-α3 collagen VI	**XG** UACC, HCT116, DLD1 colorectal cancer, **SG** B16 melanoma	~60 % reductions in tumour growth	None	[105]
TRC105 Chimeric anti-Endoglin antibody	Endoglin-BMP9	**XG** MCF7 breast cancer	Average of ~60 % reductions with cyclophosphamide	Phase II glioblastoma among others	[118]
MCAM antibody	MCAM-Netrin1	**XG** SW1990 pancreatic cancer, A375 melanoma	~70 % reductions in tumour growth when combined with bevacizumab	None	[110]

***XG*** xenograft, ***AG*** allograft, ***SG*** syngeneic, ***OT*** orthotopic, ***G*** genetic (spontaneous)

One of the major problems with therapeutic antibodies are the high costs associated with them. These costs are attributed to the expense in manufacturing and putting them through clinical trials. Future strategies may instead include the use of vaccinations using recombinant proteins of certain receptors or ligands, resulting in antibodies being raised against this target in the body rather than being administered. The quantity of recombinant protein used would be a fraction of that which is needed in antibody therapy. Such vaccinations as described previously for VEGF and FGF-2 could perhaps include recombinant proteins containing a number of different regions or epitopes from proteins involved in angiogenesis. By fusing domains of different proteins together this produces a new chimeric protein that could be seen as non-self and will most likely result in better immune responses, while still containing regions identical to the wild type proteins that the immune system can recognize. The use of epitopes in vaccines that are already known to give good anti-angiogenic blocking antibodies would be a good strategy. These epitopes in interacting regions are more likely to be immunogenic and are also solvent exposed, facing out into the environment allowing better accessibility for antibody recognition. Other advantages in the vaccine approach would be the creation of memory B cells that could be activated with tumour reoccurrence [125].

## Future of anti-angiogenics

There is recent evidence that other routes to tumour vascularisation exist, such as the ability of tumour cells to hijack existing vasculature, known as vessel co-option. β1 integrins are thought to be involved in vessel co-option in brain metastases, when β1 was blocked or deleted in mouse models, tumour cells could no longer adhere to the vascular basement membrane reducing metastasis development [126]. The role of the axon guidance molecule L1CAM has also been linked to vessel co-option in brain metastasis allowing cells to spread along capillaries [127]. Likewise the emerging role of endothelial progenitor cells recruited from the bone marrow aiding tumour angiogenesis and vasculogenesis is also another point to consider [128]. Elucidating the molecular mechanisms and protein interactions required for both of these events will likely lead to the development of therapies against them.

A major problem in cancer research is the lack of useful animal models. Animal models of cancer have been developed to give fast growing tumours to permit experimentation within an acceptable time frame. Such tumours are very different from real human cancers that are often heterogeneous and develop over long periods of time. It is clear that many anti-angiogenic agents have had preclinical success in these mouse models but this rarely translates to the clinic, there is evidently a growing need for new models that better mimic tumours seen in patients especially in metastatic disease [129].

Differences in homology of certain targeting molecules between human and mouse and the lack of cross reactive antibodies are also a limiting factor for preclinical models. The generation of humanised mouse models may be of benefit as has been achieved for VEGFA and endoglin [130] [131]. With the recent advances in genomic editing technology such as clustered regularly interspaced short palindromic repeats (CRISPR) this will likely lead to the development of more humanised mouse models [132].

Anti-angiogenic therapy was originally hailed as a blanket approach that could be used against any solid tumour; however evidence suggests that the expression profile of vasculature between different tumour types can be diverse. The differential expression of novel proteins has been shown in tumour endothelium from lung and colorectal cancer [133, 134]. Differences have even been shown between the vasculature of breast cancers of the same type, where two subtypes could be made by similarities in clusters of gene expression [135]. With advances in personalised medicine and whole transcriptome sequencing, it is not implausible to imagine a future therapy strategy that targets against various pro-angiogenic processes being utilised by a particular patient’s tumour vasculature. Targeting multiple proteins in combination with VEGF blockade, especially those thought to be important in VEGF resistance, such as Ang2 and FGF2 will likely result in better patient outcomes. With the discovery of more pro-angiogenic interactions that are important in tumour formation, we will likely gain a larger range of targets in our arsenal against cancer.
